# Anaphylactic reactions after COVID-19 vaccination in Germany 

**DOI:** 10.5414/ALX02374E

**Published:** 2023-03-31

**Authors:** Immanuel Barth, Karin Weißer, Dominique Gaston-Tischberger, Vera Mahler, Brigitte Keller-Stanislawski

**Affiliations:** 1 Division Safety of Medicinal Products and Medical Devices, and; 2 Division Allergology, Paul-Ehrlich-Institut, Langen, Germany; * The authors contributed equally to the manuscript

**Keywords:** anaphylaxis, COVID-19 vaccines, reporting rate, sex, 1^st^ dose, time interval, revaccination, follow-up survey, allergy test, pathomechanism

## Abstract

Abstract. For the COVID-19 vaccines used in Germany, severe allergic (anaphylactic) reactions after vaccination have been reported in very rare cases. While Comirnaty and Spikevax are mRNA vaccines, Vaxzevria and Jcovden comprise vector vaccines, and Nuvaxovid a recombinant spike protein vaccine. The reporting rate of anaphylaxis after mRNA vaccination was higher in females receiving their first vaccination dose, with 0.97 and 1.12 reports per 100,000 vaccinations for Comirnaty and Spikevax, respectively, compared with vaccinated males and subsequent vaccinations. The Paul-Ehrlich-Institut (PEI) investigated 106 responses of 321 cases of confirmed anaphylactic reactions concerning subsequent allergy testing and revaccination with a COVID-19 vaccine. The collected data indicate that only a small proportion of cases (22%) were IgE-mediated reactions. A large proportion (73%) of patients could be revaccinated under precautionary measures without recurrence of anaphylaxis. The pathomechanism of the majority of anaphylactic reactions remains unclear and should be investigated in further studies.

## Background 

The incidence of anaphylactic reactions after vaccination of adults and children varies between 0.2 and 13 cases of anaphylaxis per 1,000,000 vaccinations, depending on the study, study design, and vaccine (prior to COVID-19), although the estimates may be considered uncertain due to the overall small number of cases [1, 3, 4, 10, 12, 13, 19, 21]. 

While no anaphylactic reactions were observed in the pivotal trials for mRNA vaccines against COVID-19, the first cases of anaphylactic reactions after vaccination with Comirnaty (BioNTech Manufacturing, Mainz, Germany) and Spikevax (Moderna Biotech, Madrid, Spain, S.L.) were reported shortly after the initiation of the vaccination campaigns in the United Kingdom and the United States [15, 20]. 

Experts initially suspected that the lipid nanoparticles (LNP) contained in the vaccine, and in particular the polyethylene glycol (PEG) contained therein in conjugated form, could be the causative agents for the observed reactions. A possible sensitization to PEG by previous use of cosmetics or drugs containing PEG was considered. Lipid nanoparticles containing PEG are similar to liposomes, which have been used pharmaceutically for many years as carriers in drug formulations. After intravenous infusion of liposomes and other polymer-containing nanoparticles, pseudoallergic complement-mediated (non-IgE-mediated) reactions (CARPA, complement activation-related pseudoallergy) have been described. Their clinical symptoms partly overlap with those of anaphylaxis [7, 14, 22]. CARPA-mediated reactions involve complement system activation, as well as stimulation and secretion of further mediators (histamine, tryptase, platelet-activating factor, thromboxane A2, leukotrienes, cytokines, proteases, and prostaglandins) to counteract potentially harmful agents. The vector vaccines Vaxzevria (AstraZeneca AB, Sodertalje, Sweden) and Jcovden (Janssen-Cilag International NV, Beerse, Belgium) and the protein-based vaccine Nuvaxovid (Novavax CZ a.s., Jevany, Czechia) contain small amounts of polysorbate 80 as an excipient, which also has PEG moieties in the molecule. Other vaccines also contain polysorbate 80 as a stabilizer (e.g., influenza, hepatitis A, or HPV vaccines such as Fluarix, Havrix, Gardasil) [9], but reports of IgE-mediated reactions to polysorbates in drugs are an absolute rarity overall. 

Together with the Association of German Allergists (AeDA), the German Society for Allergology and Clinical Immunology (DGAKI), the Network of Severe Allergic Reactions (NORA), the German Dermatological Society (DDG), the Robert Koch Institute, and the Paul-Ehrlich-Institut (PEI), a flow chart “Procedural approach in case of positive allergy history prior to COVID-19 vaccination” was created to help vaccinators deal with the allergic history of vaccinees [18, 24]. 

The suspected cases of anaphylaxis after COVID-19 vaccination in Germany reported to the PEI, until and including March 31, 2022, are presented below. PEI also conducted a systematic follow-up of all cases meeting the Brighton Collaboration case definition of anaphylaxis in May to June 2022 (see “Materials and methods” below) to inquire about the outcome of allergy testing and the tolerability of further vaccination. The results of the follow-up survey of affected vaccinated individuals and treating physicians are summarized. 

## Materials and methods 

In Germany, according to section 6 subsection 1 sentence 1 no. 3 of the Infection Protection Act (IfSG), the suspicion of health impairment exceeding the usual level of a vaccination-related adverse reaction must be reported by patient identification. The notification is made by the physician to the public health department. According to section 11 subsection 4 IfSG, the health offices are obliged to submit the reported suspected cases to the competent federal authority and the competent state authority, the PEI, in pseudonymized form. In addition, physicians and pharmacists have professional reporting obligations to the respective Drug Commission, which in turn reports to the PEI. Independently of this, there is the possibility of direct reporting to the PEI by the public. All reported suspected cases are archived in a relational database of the PEI and encoded according to the legally binding standard. A relational database is a type of database that allows storage and access to interconnected data points. Potential reports of anaphylaxis after COVID-19 vaccination from Germany were identified using a search query with the following terms from the Standardized Medical Dictionary for Regulatory Activities (MedDRA, [11]) (Preferred Term level: Anaphylactic reaction (10002198), Anaphylactic shock (10002199), Anaphylactoid reaction (10002216), Anaphylactoid shock (10063119), Hypersensitivity (10020751), Drug hypersensitivity (10013700)). 

The above search algorithm associated with received COVID-19 vaccine reports was conducted using the PEI database on suspected adverse drug reactions for the period December 27, 2020 (start of the vaccination campaign in Germany) to March 31, 2022 inclusive (date of database access: April 05, 2022). The results were validated by physicians in PEI and classified according to the level of diagnostic certainty, according to the Brighton Collaboration (BC) case definition [19]. Regardless of the causality of anaphylaxis, three levels of diagnostic certainty (BC levels 1 – 3) are defined based on “major” and “minor case definition criteria” (Table 1A, Table 1B). All three levels require an unexpected, sudden onset of symptoms (“sudden onset”, “unexpected”) and a rapid worsening of symptoms (“rapid progression”). 

BC level 4 does not permit a final assessment without further mandatory information. If clinical information was initially missing, an extended questionnaire was sent to the reporting person to specifically obtain further clinical information to assess diagnostic certainty. 

The reporting rate of anaphylactic reactions was vaccine-specific based on the number of anaphylactic reactions classified as BC level 1 – 3 or BC level 1 – 4 according to the vaccines administered. The PEI obtained background vaccination rate data from the Robert Koch Institute, with additional data from the IQVIA Disease Analyzer from IQVIA Commercial GmbH & Co. OHG, Munich, Germany [5] used for sex-specific stratification (for exposure determination, see [16]). 

During May and June 2022, PEI conducted an additional systematic follow-up using a separate standardized questionnaire (see “Results” section below) for all suspected case reports of anaphylaxis following COVID-19 vaccines classified as BC levels 1 – 3. By written or telephone interview with the reporting persons, the results of allergy tests and the tolerability of further COVID-19 vaccinations were added after consent of the affected vaccinated persons. 

## Results 

### Initial reports 

By March 31, 2022, 562 reports of anaphylactic reaction after COVID-19 vaccination were reported to PEI, which were rated BC levels 1 – 4 by PEI, regarding diagnostic certainty. These included 321 BC level 1 – 3 cases, of which 183 cases were BC level 1, 121 cases were BC level 2, and 17 cases were BC level 3. Among 321 BC level 1 – 3 anaphylaxis cases, 8 cases (7 after first and 1 after third Comirnaty vaccination) were reported for children and adolescents: one 7-year-old girl (BC level 2), one 15-year-old girl (BC level 2), and six 17-year-old girls (5 BC level 1, 1 BC level 2). In 557 (316 BC levels 1 – 3) reports, the vaccine was known (these are shown in Table 2); in 5 cases, the COVID-19 vaccine was not mentioned. In 383/557 (69%) of all cases and in 231/316 (73%) of cases with BC levels 1 – 3 (with known vaccine), the reaction occurred after the first vaccination. A reaction after the second vaccination was reported by 112/557 (20%) and 73/316 (23%), respectively, and after the third vaccination by 20/557 (3%) and 4/316 (1%), respectively. Dose number information was missing in 42/557 (8%) and 8/316 (3%) cases, respectively. 

Reporting rates for anaphylaxis after COVID-19 vaccination with mRNA and vector vaccines ranged from 0.19 to 0.51 for reports with BC levels 1 – 4 and from 0.07 to 0.36 for reports with BC levels 1 – 3, based on 100,000 vaccinations. Only isolated case reports are currently available for Nuvaxovid, so the reporting rate should be interpreted with caution. Reporting rates are elevated for the mRNA vaccines and for Vaxzevria in female vaccinees as well as for the first dose compared to subsequent vaccinations. 

### Time interval between vaccination and first symptoms 

Of the 321 cases of anaphylactic reactions BC level 1 – 3, who received vaccination with Comirnaty, Spikevax, Vaxzevria, Jcovden, or Novaxovid, time to onset (TTO) from vaccination was reported in 242 cases. In these 242 cases, anaphylactic symptoms occurred within 15 minutes in 64%, within 30 minutes in 82%, and within 60 minutes in 91%. An interval between the 1^st^ and 6^th^ hour, between the 6^th^ and 12^th^ hour, and between the 12^th^ and 24^th^ hour after vaccination was reported in 3% of cases each (Table 3). 

### Clinical symptoms 

The following symptoms were indicated in the reports of anaphylaxis that met the case definition: 

1. Dermatologic: urticaria (69 ×), angioedema (49 ×), Quincke’s edema (8 ×), facial swelling (37 ×), swelling of tongue (23 ×), lips (20 ×), throat (17 ×), maw (12 ×), pharynx (2 ×), larynx (3 ×), whole body (12 ×), rash (16 ×), exanthema (64 ×), erythema (65 ×), generalized symptomatology (207 ×), pruritus/itching (28 ×). 

2. Cardiovascular: circulatory instability (17 ×), hypotension (61 ×), tachycardia (98 ×), circulatory collapse (6 ×), circulatory instability (2 ×), unconsciousness (10 ×). 

3. Respiratory: dyspnea (55 ×), shortness of breath (30 ×), labored breathing (2 ×), bronchospasm (37 ×), tachypnea (76 ×), stridor (41 ×), wheezing (26 ×), throat tightness (114 ×) 

4. Gastrointestinal: abdominal pain (33 ×), nausea (23 ×), diarrhea (18 ×), nausea/sickness (95 ×). 

Note: At the PEI, reported adverse reactions are coded according to MedDRA terminology. In this process, reported reactions are coded as literally as possible. Therefore, there are different codes for similar reactions in MedDRA, e.g. Preferred Term (PT) Exanthema (MedDRA code: 10015585), PT Rash (MedDRA code: 10040913). 

### Treatment and outcome 

Of 321 anaphylaxis cases with BC levels 1 – 3, 108 (33.6%) patients received emergency treatment with epinephrine, including 70 × BC level 1, 36 × BC level 2, and 2 × BC level 3. In 268 (83.5%) cases, a cortisone preparation and/or antihistamine was administered. In 104/268 (38.8%) of these cases, a combination therapy with epinephrine was given, of which 68 × BC level 1, 34 × BC level 2, and 2 × BC level 3. In 164/268 (61.2%) cases, an exclusive therapy with a cortisone preparation and/or antihistamine was given, of which 88 × BC level 1, 64 × BC level 2, and 12 × BC level 3. 

Neither fatal outcome nor permanent damage was reported for the individual cases classified as BC level 1 – 3. 

### Follow-up 

Of 321 cases with BC level 1 – 3, PEI received 106 (33%) follow-up reports (of which 70 cases were BC level 1 (66%), 29 cases were BC level 2 (27%), 7 cases were BC level 3 (7%)). The vaccine involved was Comirnaty in 67 cases, Spikevax in 10 cases, Vaxzevria in 26 cases, Jcovden in 2 cases, and Nuvaxovid in one case. In 80/106 (76%) responses, the anaphylactic reaction had occurred after the first COVID-19 vaccination, and in 24/106 (23%) after the second vaccination (two cases lacked this information). 

The results are shown in Table 4. 

### Results of allergy testing 

Allergy testing was performed in 37/106 cases (35%) (Table 4). The allergy testing included skin tests (mainly prick tests) with the respective COVID-19 vaccine (partially diluted) and with solutions of PEG (polyethylene glycol, component of an excipient in Comirnaty and Spikevax) or polysorbate 80 (excipient in Vaxzevria, Jcovden, and Nuvaxovid). 

**Figure 1.**
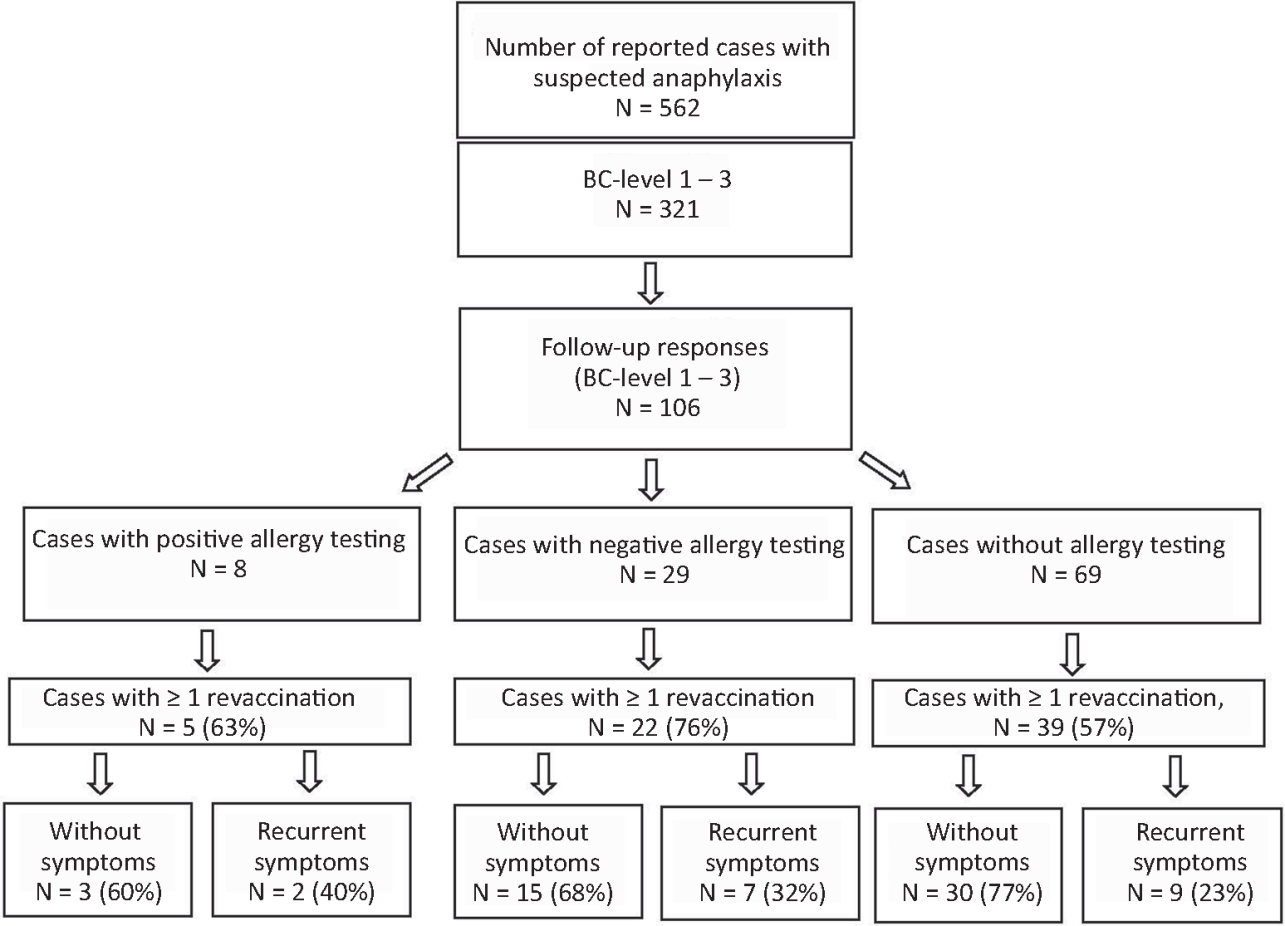
Results of follow-up of suspected anaphylactic reaction reports after COVID-19 vaccination (Table 4).

Allergy testing revealed a positive reaction to the vaccine or an ingredient in 8/37 cases (22%, BC level 1 n = 7, BC level 3 n = 1) and no reaction occurred in 29 cases (Table 4) (Figure 1). 

Of the 5 Comirnaty cases that tested positive, 3 patients reacted to the vaccine itself and 2 patients reacted to a PEG solution. One Spikevax-vaccinated individual, who tested positive, and 1 of 2 Vaxzevria-vaccinated individuals who tested positive showed a positive skin reaction to the respective vaccine. In 1 case, the exact test results are not available. 

In 69/106 (65%) follow-up cases, no allergological investigation was performed (47 after Comirnaty, 5 after Spikevax, 15 after Vaxzevria, and 1 case each after Jcovden and Nuvaxovid) (Table 4). The reasons for this were diverse (e.g., lack of information about availability for allergy testing, long distance to the allergy center up to self-diagnosis (“I am sure it was PEG”)). 

### Repeat COVID-19 vaccination 

Overall, of the 106 individuals with an anaphylactic reaction after COVID-19 vaccination who participated in follow-up, 66 (62%) individuals received at least 1 additional COVID-19 vaccination (41 individuals reported 1 re-vaccination, 22 individuals reported 2 re-vaccinations, and 3 individuals reported 3 additional vaccinations). 

The re-vaccinations were carried out after allergy testing in 27/66 (41%) and without prior allergy testing in 39/66 (59%). The proportion of re-vaccinated individuals in the group of tested (27/37, 73%) is higher than in the group of non-tested (39/69, 57%). More than one-third of the vaccinated (40/106, 38%) refrained from further vaccination (Table 4) (Figure 1). 

24 of 66 (36%) re-vaccinated individuals received one or more re-vaccinations with the same vaccine as “re-challenge”. Of these individuals, 9/24 (38%) had previously undergone allergy testing. In this case, 8/9 (89%) tested negative and 1/9 (11%) tested positive. In this case (BC level 3), a positive reaction was noted in the skin test with the (diluted) vaccine, but it was very mild and of delayed reaction pattern after 3 – 4 hours. Here, another vaccination with the same vaccine (Comirnaty) was performed despite a positive reaction in the skin test. This person reacted again to the second vaccination with Comirnaty, administered without particular precautions. However, the symptoms were reported as milder compared with the first vaccination. A third vaccination with Comirnaty was subsequently tolerated with almost no symptoms. 

3 of the 8 individuals who tested negative received re-exposure to the same vaccine and reacted again. In 1 case, after initial vaccination with Comirnaty, with a temporal interval (time to onset, TTO) of 20 minutes, a symptom complex was described with dyspnea, retrosternal tightness, positive shock index, tension headache, and generalized itching, numbness, and edematous swelling of the hands and face. This person, despite a negative skin prick test to the vaccine, reacted immediately with a feeling of thoracic pressure and tightness during two further fractionated follow-up vaccinations with Comirnaty in each case, but the symptoms did not increase as on the first occasion. No dyspnea occurred, and no further medical treatment was necessary. In the second case, after the first vaccination with Spikevax, anaphylaxis BC level 2 occurred, requiring hospitalization. After the skin prick test for PEG solutions as well as for all vaccine samples was negative, a fractionated second vaccination with Spikevax was performed. Already during the administration, a recurrent reaction with the symptoms of chills and nausea and, in the course, fever appeared. In the third case (first vaccination with Vaxzevria), the second Vaxzevria vaccination was followed by a recurrent immediate reaction with dyspnea, tingling, orthostatic circulatory reaction, with similar intensity as to the first vaccination. The previously performed allergy testing did not include testing for polysorbate 80. It only included negative skin prick testing for Movicol (PEG). The subject again reported the above symptoms on a third vaccination with Comirnaty, which was also described in the past medical history to other drugs (e.g., Simponi, which tested negative in a skin prick test). Chronic spontaneous urticaria with a concomitant urticaria factitia is known since 2018. 

The other 5 of 8 individuals who tested negative tolerated the re-challenge well without a recurrent reaction. 

42 of 66 (64%) re-vaccinated individuals received another COVID-19 vaccine (this includes “other mRNA vaccine”). 18 of the 42 (43%) subjects had prior allergy testing. In this process, 14/18 (78%) individuals tested negative. 

Of these 14 subjects who tested negative 10 (71%) tolerated the subsequent other vaccine without problems (Table 4) (Figure 1). In 3 cases with previous Comirnaty vaccination, a recurrent but weaker immediate reaction occurred despite a negative test. Only in 1 of the 3 cases, a premedication had been administered before the new vaccination. In 1 case with Spikevax first vaccination (BC level 2, after 30 minutes of facial swelling, lip/tongue swelling, dysphagia, tingling, shortness of breath, flushing symptoms) reactions occurred again (facial swelling, dysphagia, no dyspnea, TTO 20 minutes), despite a negative skin test for second vaccination with Vaxzevria. On third vaccination with Comirnaty, under antihistamine prophylaxis, unspecific complaints were reported (nausea, dizziness, increase in tinnitus 10 minutes post vaccination (p.v.), and chest tightness 1 hour p.v.). 

Three of the 4 individuals who tested positive subsequently received Spikevax (1), Vaxzevria (1), or Jcovden plus subsequent Nuvaxovid (1) after initial vaccination with Comirnaty. In each case, the re-vaccinations were well tolerated. In the 4^th^ case, after initial vaccination with Vaxzevria (5 minutes p.v.), the vaccinee experienced swelling of the throat, difficulty swallowing (unable to swallow), tingling of the face, throat, palate, and tachycardia. During the second vaccination with Comirnaty, circulatory collapse with palpitations and respiratory distress occurred. The previous skin prick test for PEG was negative and positive for Vaxzevria itself. 

In summary, 5 of 8 individuals who tested positive received re-vaccinations, 1 with the same vaccine (Comirnaty-Comirnaty-Comirnaty) and 4 with a different vaccine (Comirnaty-Spikevax, Comirnaty-Vaxzevria, Comirnaty-Jcovden, Vaxzevria-Comirnaty; see above). Two subjects (Comirnaty-Comirnaty-Comirnaty and Vaxzevria-Comirnaty^®^) reacted with a similar reaction as described above. In 1 of these 2 individuals (Comirnaty-Comirnaty-Comirnaty), an IgE-mediated reaction is unlikely due to the described decrease in symptom severity with further applications of the same vaccine. Three out of 5 subjects could be vaccinated with a different vaccine without any problems (Figure 1). 

Special precautions were taken to re-vaccinate 34/66 cases (52%), including 2 of 5 who tested positive, 18 of 22 who tested negative, and 14 of 39 who had not received an allergy test at all. Precautions included increased emergency preparedness (e.g., venous line (10), vaccination under inpatient conditions (5), extended observation time (4), fractionated vaccination (5), prophylactic administration of an antihistamine (6), a cortisone preparation (10), both together (2)). In contrast 28 of 66 persons were re-vaccinated without special precautions (in another 4 cases, there is no information on this). Five of these 28 individuals (18%) had previously received allergy workup. 

## Discussion 

The reporting rates of anaphylactic reactions for COVID-19 vaccines authorized in Germany are significantly lower than 1 case per 100,000 vaccinations. Thereby, the reporting rate is comparatively higher after the first dose and in females. The dominance of females, in terms of reports of anaphylaxis in adults, has also been noted with other vaccines in other studies [24]. The reporting rate of anaphylactic reactions in Germany is comparable to that of other passive surveillance systems: The CDC (Centers for Disease Control and Prevention) reported initial U.S. reporting rates BC levels 1 – 3 of 0.47 per 100,000 vaccinations with Comirnaty and 0.25 per 100,000 vaccinations with Spikevax [20] in the period from December 14, 2020 to January 18, 2021. Presumably, first-time vaccinations were predominantly given during this period. For Nuvaxovid, 2 cases of anaphylaxis, meeting the case definition of BC levels 1 – 3, were reported to the PEI by the time of the evaluation. Therefore, the calculation of a reporting rate is associated with large uncertainties. 

In general, monitoring of at least 15 minutes after COVID-19 vaccination is recommended according to the SmPC. In 64% of reports (BC levels 1 – 3), first symptoms occurred during the recommended monitoring interval, and 82% of reported cases occurred within a 30-minute window. 

Allergy testing was performed in 1/3 (35%) of follow-up cases of anaphylactic reactions. Among those tested, 22% (8 cases) reacted positively. That is, in 78% of all tests, no (skin) reaction to the vaccine or components therein was observed. Only 2 of 25 tested persons, who previously showed an anaphylactic reaction after mRNA vaccination, reacted to a PEG solution in the skin test. No positive test result to polysorbate was reported. 

The results indicate that the majority of reactions were not due to IgE antibodies to the antigen or excipients such as PEG or polysorbate 80. The challenge of performing allergy testing with non-commercial/non-approved test preparations has been pointed out before elsewhere [2, 25]. 

In 7/8 individuals who tested positive, anaphylaxis occurred after the first vaccine dose. Furthermore, this, as well as the overall majority of reports after first vaccination, argues against sensitization by the vaccine, e.g., to an ingredient (e.g., PEG/polysorbate 80), and rather for a pseudoallergic reaction or pre-existing hypersensitivity. 

Fortunately, 62% of all persons with anaphylaxis after initial vaccination could be re-vaccinated. The fact that the proportion of re-vaccinated persons in the allergy-tested group (73%) is higher than in the non-tested group (57%) could be due to the fact that the desire for vaccination was higher in the persons with allergy testing than in the non-tested persons or that the testing performed increased the willingness for re-vaccination. It is noteworthy that 15 individuals (without prior testing) were re-vaccinated with the same vaccine. Of the 15, 6 individuals reported repeat adverse reactions. 

Eight individuals with negative allergy test results were re-vaccinated with the same vaccine; of these, 3 individuals reported reactions after re-vaccination, although less pronounced than after initial vaccination, possibly related to premedication. Overall, 14 of 18 re-vaccinated subjects, with recurrent adverse reactions, reported a decrease in reaction severity. Unfortunately, whether these cases may represent a pseudoallergic reaction has not been investigated. Pseudoallergy is characterized by an immediate systemic reaction with symptoms similar to anaphylaxis, but the release of mediators is not mediated by IgE. Pseudoallergy is often triggered by the first dose of a drug that stimulates mast cells and basophils to degranulate. Pseudoallergy does not trigger an antigen-specific immune response but rather histamine release, activation of the complement system, atypical synthesis of eicosanoids, and inhibition of bradykinin degradation. In contrast to IgE-mediated reactions [26], an attenuation of the reaction is observed with repeated applications [14]. Mechanisms include particle-induced complement activation (CARPA [14, 22]) or direct mast cell activation [26]. Warren et al. [23] reported a case series of patients with anaphylactic reaction to COVID-19 mRNA vaccination, who were negative in skin prick test to PEG (11/11) and to the respective vaccine (10/11), but showed a positive BAT test to PEG (10/11) and to the respective vaccine (11/11). No individual was found to have anti-PEG IgE; instead, high levels of anti-PEG IgG antibody were measured, indicating a non-IgE-mediated response. Lim et al. [8] also measured elevated biomarkers in 3 patients with anaphylaxis after Comirnaty vaccination, indicating a pseudoallergic reaction (anti-PEG IgG/IgM, C3a). This is consistent with results of retrospective studies indicating that anaphylactic reactions following COVID-19 vaccination, in the majority of individuals studied, do not appear to be due to IgE-mediated immediate allergic reactions. Affected patients could mostly be re-vaccinated with low risk after allergy testing [6, 17, 23]. 

The fact that more than half of the individuals who were re-vaccinated with the same vaccine in our follow-up tolerated re-vaccination without adverse reactions, as well as the fact that, on the other hand, anaphylactic reactions also occurred to different COVID-19 vaccines (up to three vaccines completely different in their composition) also does not substantiate the specificity of the initial reaction. Differentially, in some cases with clinical symptoms upon re-vaccination, anxiety reactions cannot be excluded. 

Overall, the follow-up shows that specific allergic reactions to the vaccines or components seem to be a rare exception and that re-vaccination was possible without problems in the vast majority of cases with both the same and an alternative vaccine. 

In the follow-up conducted by the PEI, data could be collected from 106 persons, and results of allergy testing are known from 37 persons; this is a number of cases comparable to other investigations. Standardized questionnaires were used to assess diagnostic confidence and for follow-up. Allergy test results were transmitted as a copy. 

A weakness of the follow-up is that in only one third of the reported cases of anaphylaxis further information was submitted; therefore, it is unclear how representative the results are. The product information for COVID-19 vaccines indicates that individuals who have developed an anaphylactic reaction after vaccination should not be re-vaccinated with the vaccine. Therefore, it can be assumed that especially those persons who have received an allergy examination or have been vaccinated again, have had a particularly high interest in a further vaccination and a completion of the vaccination protection despite the notice in the product information. Since in this retrospective study different test preparations and methods were used for allergy testing according to best available medical practice, neither false positive nor false negative test results can be excluded in individual cases. 

## Conclusions

- The PEI data do not indicate a significantly higher risk of anaphylactic reactions after the COVID-19 mRNA vaccines Comirnaty and Spikevax or the adenovirus-based vector vaccines Vaxzevria and Jcovden compared to other vaccines.
- A second COVID-19 vaccination was asymptomatic in a majority (73%) of patients with anaphylactic reaction after COVID-19 vaccination, including 14 individuals receiving the same vaccine.
- The test results (8 positives in 37 individuals tested, 22%) and the prevailing low-risk re-vaccination (also with the same vaccine) indicate that only a small proportion of cases are IgE-mediated reactions.
- The pathomechanism of the majority of anaphylactic reactions is unknown and should be decisively investigated in further studies.

## Funding 

None. 

## Conflict of interest 

The authors declare no conflict of interest. 

## Disclaimer 

The positions expressed in this work reflect the expert opinion of the authors and may not be interpreted or quoted as if they were given on behalf of the responsible national higher federal authority, the European Medicines Agency or their committees or working groups or represent their position. 

**Table 1A.** Description of symptoms typical of anaphylaxis according to organ system affected and classification into major and minor criteria [19].

	Dermatological	Cardiovascular	Respiratory	Gastrointestinal	Laboratory
MAJOR	- Generalized* urticaria or generalized erythema - Angioedema - Generalized pruritus and skin rash	- Measured hypotension systemic RR < 90 mmHG or decrease > 30% from baseline - Clinical diagnosis of an uncompensated shock, at least 3 out of 4: • Tachycardia • Capillary refill time** > 3 seconds • Reduced central pulse volume • Decreased level of consciousness or loss of consciousness	- Bilateral wheeze (bronchospasm) - Stridor - Upper airway swelling (lips, tongue, throat) - Respiratory distress, at least 2 out of 5: • Tachypnea • Increased use of the accessory respiratory muscles • Recession • Grunting • Cyanosis		
MINOR	- Generalized pruritus without skin rash - Generalized prickle sensation - Localized injection site urticaria - Red and itchy eyes	- Reduced peripheral circulation, at least 2 of 3: • Tachycardia • A capillary refill time** of > 3 seconds without hypotension • A decreased level of consciousness	- Persistent dry cough - Hoarse voice - Difficulty breathing without wheeze or stridor - Sensation of throat closure - Sneezing - Rhinorrhea	- Diarrhea - Abdominal pain - Nausea - Vomiting	- Elevated mast cell tryptase

*Generalized = affecting at least two parts of the body (injection site counts); **Capillary refill time: Capillary refill time is defined as the time it takes for color to return to the outer capillary bed after pressure has been applied to cause bleaching. In humans, a recapitalization time greater than 3 seconds indicates reduced peripheral blood flow and may be indicative of cardiovascular or respiratory dysfunction.

**Table 2.** Number of reports of anaphylaxis (BC levels 1 – 4) from Germany to the Paul-Ehrlich-Institut according to COVID-19 vaccines and reporting rates per 100,000 vaccinations overall, in women and in men (reporting period: December 27, 2020 to March 31, 2022; 5 case reports without COVID-19 vaccine designation are not included here).

	Comirnaty		Spikevax		Vaxzevria		Jcovden		Nuvaxovid		Total amount
	BC 1 – 3	BC 1 – 4	BC 1 – 3	BC 1 – 4	BC 1 – 3	BC 1 – 4	BC 1 – 3	BC 1 – 4	BC 1 – 3	BC 1 – 4	
Dose 1	167	274	15	36	44	60	4	9	1	5	231/384
Dose 2	66	102	4	5	2	2	–	1	1	1	73/111
Dose 3	3	11	1	9	–	–	–	–	–	–	4/20
k.A.	7	32	–	6	–	3	1	1	–	–	8/42
Total amount	243	419	20	56	46	65	5	11	2	6	316/557
**Reporting rate**											
Total	0.19	0.33	0.07	0.19	0.36	0.51	0.14	0.30	2.5	7.4	
Female	0.32	0.52	0.12	0.29	0.66	0.93	0.08	0.40	4.5	13.5	
Male	0.05	0.11	0.02	0.11	0.08	0.09	0.17	0.25	–	–	
**Reporting rate dose 1**											
Total	0.37	0.60	0.29	0.71	0.47	0.65	0.11	0.25	1.3	6.2	
Female	0.62	0.97	0.56	1.12	0.88	1.21	0.08	0.32	3.5	17.6	
Male	0.08	0.18	0.04	0.31	0.10	0.12	0.13	0.21	–	–	

Comirnaty und Spikevax are COVID-19 mRNA vaccines (nucleoside modified); Vaxzevria (COVID-19 vaccine (ChAdOx1-S [recombinant])) and Jcovden (COVID-19 vaccine (Ad26.COV2-S [recombinant])) are adenovirus-based vector vaccines; Nuvaxovid is a recombinant, adjuvanted protein vaccine (COVID-19 vaccine [recombinant], adjuvanted).

**Table 3.** Time to onset (TTO) between vaccination and onset of first symptoms of anaphylaxis (BC levels 1 – 3) in minutes (min) or hours (h).

TTO	Number of anaphylaxis cases	Percentage	Cumulative
< 15 minutes	155	64.0%	64.0%
15 – 30 minutes	44	18.2%	82.2%
30 – 60 minutes	20	8.3%	90.5%
1 – 6 hours	8	3.3%	93.8%
6 – 12 hours	8	3.3%	97.1%
12 – 24 hours	7	2.9%	100%
Total	242	100%	100%

TTO = Time to onset.

**Table 4.** Results of the follow-up of all cases with BC level 1 – 3.

Vaccine of the initial report	Comirnaty			Spikevax			Vaxzevria			Jcovden			Nuvaxovid			All (Total)		
Follow-up cases with feedback	67			10			26			2			1			106		
**Allergological clarification**																		
Vaccine/component allergy test?	**pos**	**neg**	**nd**	**pos**	**neg**	**nd**	**pos**	**neg**	**nd**	**pos**	**neg**	**nd**	**pos**	**neg**	**nd**	**pos**	**neg**	**nd**
	5	15	47	1	4	5	2	9	15	–	1	1	–	–	1	8	29	69
on whole vaccine	3	7	–	1	3	–	1	3	–	–	–	–	–	–	–	5	13	–
on PEG	2	8	–	–	3	–	–	7	–	–	–	–	–	–	–	2	18	–
on polysorbate	–	4	–	–	–	–	–	6	–	–	–	–	–	–	–	–	10	–
Anaphylaxis^(X)^ after dose 1	4	11	31	1	3	4	2	8	14	–	1	1	–	–	–	7	23	50
Anaphylaxis after dose 2	1	4	15	–	1	1	–	1	1	–	–	–	–	–	1	1	6	17
Anaphylaxis after dose 3	–	–	–	–	–	–	–	–	–	–	–	–	–	–	–	–	–	–
**Revaccination**																		
Revaccinated	4	11	22	–	4	4	1	6	13	–	1	–	–	–	–	5	22	39
Under special precautions	1	9	9	–	4	2	1	5	3	–	–	–	–	–	–	2	18	14
With the same vaccine	1	6	13	–	1	2	–	1	–	–	–	–	–	–	–	1	8	15
Recurrent reaction to the same vaccine	1	1	4	–	1	2	–	1	–	–	–	–	–	–	–	1	3	6
With another vaccine	3	5	9	–	3	2	1	5	13	–	1	–	–	–	–	4	14	24
Recurrent reaction to other vaccine	–	3	1	–	1	–	1	–	2	–	–	–	–	–	–	1	4	3

Comirnaty und Spikevax are COVID-19 mRNA vaccines (nucleoside modified); Vaxzevria (COVID-19 vaccine (ChAdOx1-S [recombinant])) and Jcovden (COVID-19 vaccine (Ad26.COV2-S [recombinant])) are adenovirus-based vector vaccines; Nuvaxovid is a recombinant, adjuvanted protein vaccine (COVID-19 vaccine [recombinant], adjuvanted). (X) in two cases no information on the vaccination dose.

**Table 1B.** Minimum requirements for the diagnostic certainty levels (levels 1 – 4) according to the Brighton Collaboration (BC) case definition [19].

Level 1	Major criterion dermatological + major criterion cardiovascular or respiratory
Level 2 (three alternatives)	Major criterion cardiovascular + major criterion respiratory
	Major criterion cardiovascular or respiratory + minor criterion other organ system
	Major criterion dermatological + minor criterion cardiovascular or respiratory
Level 3	Minor criteria from three different organ systems, of which at least one is cardiovascular or respiratory
Level 4	Reports of anaphylactic reactions without sufficient information on the symptoms and progression, so it is not possible to assess the diagnostic certainty
